# Clinical significance of *Mycoplasma pneumoniae* specific IgM titer in children hospitalized with *Mycoplasma pneumoniae* pneumonia

**DOI:** 10.1186/s12879-022-07456-6

**Published:** 2022-05-16

**Authors:** Soojeong Choo, Seo-Hee Kim, Eun Lee

**Affiliations:** Department of Pediatrics, Chonnam National University Hospital, Chonnam National University Medical School, Jebong-ro, Dong-gu, Gwangju, 61469 Republic of Korea

**Keywords:** Children, IgM, *Mycoplasma pneumoniae*, Pneumonia

## Abstract

**Background:**

The present study aimed to identify the clinical significance of *Mycoplasma pneumoniae* (MP)-specific immunoglobulin M (IgM) titer, in addition to a diagnosis of MP infection, in children with MP pneumonia.

**Methods:**

This study was performed in 155 children hospitalized with MP pneumonia. The clinical features and laboratory and radiographic findings on admission in children with positive or negative MP-specific IgM titers were retrospectively reviewed from the electronic medical records.

**Results:**

The mean age of the included children was 6.0 years, and 118 (76.1%) of the children were positive for MP-specific IgM. A longer duration between symptom onset and admission (adjusted odds ratio [aOR] 1.47, 95% confidence interval [CI] 1.24–1.75), longer duration of symptoms during the illness (aOR 1.15, 95% CI 1.02–1.30), and development of extra-pulmonary manifestations (aOR 9.16, 95% CI 1.96–42.81) were significantly associated with a positive MP-specific IgM titer. Serum lactate dehydrogenase levels (aOR 1.00, 95% CI 1.00–1.01) and pneumonic infiltration involving > 50% of the total lung volume on chest radiography (aOR 4.68, 95% CI 1.12–19.55) were associated with positive MP-specific IgM in children with MP pneumonia. A poor response to stepwise treatment for MP pneumonia was more common in children with a positive MP-specific IgM titer than those with a negative MP-specific IgM titer on admission.

**Conclusions:**

A positive MP-specific IgM titer at diagnosis of MP pneumonia may partially suggest an exaggerated immune response with a higher disease burden compared to children with MP pneumonia with a negative MP-specific IgM titer.

## Background

Diverse respiratory viruses, *Mycoplasma pneumoniae* (MP) and other bacteria can cause pneumonia in children [1, 2]. In children with community-acquired pneumonia (CAP), early identification of the causative respiratory pathogens affects treatment strategies, including the choice of appropriate antibiotics and/or immune-modulatory drugs, and even prognosis [2]. MP is one of the most common causes of CAP in children, accounting for up to 40% with differences in age and geographic regions [1]. Although the clinical course of MP pneumonia is known to be mild [1], some MP pneumonia cases have severe clinical courses with complications [3, 4]. In recent years, the prevalence of refractory MP pneumonia in children, characterized as a poor response to treatment with a high prevalence of complications, has been increasing [5]. To prevent the development of complications associated with MP pneumonia in children and decrease the disease burden, early diagnosis and early therapeutic interventions for MP pneumonia are important.

However, the diagnosis of MP pneumonia in children is sometimes delayed due to false negative test results for MP-specific immunoglobulin M (IgM), especially in the early phase of MP infection, and variable detection rates of polymerase chain reaction (PCR) for MP due to diverse factors such as patient age, cooperation during the sample collection, and sampling sites [6–8]. Therefore, early etiologic identification in MP pneumonia has been challenging.

Identification of MP-specific IgM titers is the most widely used serologic test to diagnose MP infection. However, a single negative MP-specific IgM titer cannot exclude MP infection. Although a MP-specific IgM titer usually shows seroconversion during the first 7 days following symptom onset, the MP-specific IgM titer is diverse even within this period of illness due to MP infection [9]. Furthermore, factors associated with a positive MP-specific IgM titer, excluding the approximate duration of symptom onset, have not been elucidated.

This study aimed to compare the clinical features and laboratory findings between children with a positive MP-specific IgM titer and those with a negative MP-specific IgM titer on admission in children hospitalized with MP pneumonia. In addition, we elucidated the associated factors with a positive MP-specific IgM titer at the time of admission in children hospitalized with MP pneumonia.

## Methods

### Study participants

This retrospective study comprised 155 hospitalized children with MP pneumonia between May 2019 and February 2020. The inclusion criteria were patients who meet the following items: (i) confirmed MP infection using both MP serologic (Chorus MP IgM ELISA, Diesse Diagnostica, Senese, Siena, Italy) and PCR (*M. pneumoniae* Real-Time PCR kit, Slan; Biocore, Seoul, South Korea) tests during the illness, (ii) identification of seroconversion of an MP-specific IgM titer in patients with an initial negative MP-specific IgM titer, which confirmed MP infection when combined with positive results on PCR tests, and (iii) proven MP pneumonia based on recent history of the presenting illness, physical examination, and chest radiographic findings in previously healthy children. The exclusion criteria comprised patients with immunosuppressive diseases, taking immunosuppressive drugs, and with underlying diseases associated with any chronic lung diseases or those with recurrent respiratory infections, defined as more than two episodes of respiratory infection in the past 2–3 months. The clinical features and laboratory and radiographic findings in children with a positive MP-specific IgM titer at the time of admission and those with a negative MP-specific IgM titer at the time of admission were retrospectively reviewed using electronic medical records. This study was approved by the Institutional Review Board (IRB) of Chonnam National University Hospital, which waived the need for informed consent (IRB no. CNUH-2019-261).

### Definitions

For the MP-specific IgM titer, identified using the ELISA method, the cut-off values for interpreting MP infection status were assessed in accordance with the manufacturer’s instructions, as follows: positive (IgM, > 1.1) and negative (IgM, < 0.9). The extra-pulmonary manifestations associated with MP pneumonia in the present study included skin rash; acute hepatitis, which was reflected in elevated aspartate aminotransferase (AST) and alanine aminotransferase (ALT); and pulmonary thromboembolism.

The patients with MP pneumonia were treated using the following strategies. First, patients with MP pneumonia were treated with macrolides combined with intravenous methylprednisolone (1–2 mg/kg/day; maximum 30 mg/dose) in severe cases to reduce the excessive immune response [10, 11]. If there was no response to the first-line therapy within 3–5 days, ciprofloxacin or tetracyclines were added to the treatment for patients with macrolide-resistant MP pneumonia. If there was no response to the second-line antibiotic therapy after 3–5 days, methylprednisolone (10–15 mg/kg/day) pulse therapy was administered for 3 consecutive days.

The response to treatment for MP pneumonia was classified into four groups, based on the response to the aforementioned stepwise treatment for MP pneumonia: good response, slow response, no response, and progression [11]. A good response was defined as an improvement in respiratory symptoms and/or plain chest radiography findings within 2–3 days of applying the stepwise treatment for MP pneumonia; a slow response was defined as an improvement in respiratory symptoms and/or chest radiography findings within 1 week, but not within 2–3 days; no response was defined as the absence of improvement; and progression was defined as progression in respiratory symptoms and/or chest radiography findings even after 1 week of applying the stepwise treatment for MP pneumonia. The four groups are divided into good and poor response groups, where the poor response group included patients with slow response, no response, and progression to stepwise treatment for MP pneumonia. The severity of pneumonia based on the extent of pneumonic infiltration on the chest x-ray at the time of admission was defined as follows: mild as pneumonic lesion involving < 1/3 of the total lung volume; moderate as involvement of more than 1/3, but less than 1/2 of the total lung volume; and severe as involvement of more than 1/2 of the total lung lesion.

### Microbiologic investigations

Respiratory virus co-infection was investigated using a PCR assay kit (Anyplex II RV16 detection kit, Seegene, Seoul, South Korea) with nasopharyngeal swab samples for the common 16 respiratory viruses, including adenovirus, bocavirus, corona viruses OC43, 229E, and NL63, enterovirus, influenza viruses A and B, human metapneumovirus, parainfluenza viruses 1–4, respiratory syncytial viruses A and B, and rhinovirus. A total of 73 children in the study also underwent a pneumobacter PCR (Allplex Pneumobacter Assay, Seegene, Seoul, South Korea) assay to identify combined bacterial coinfection with *Hemophilus influenzae*, *Streptococcus pneumoniae*, *Chlamydia pneumoniae*, *Bordetella pertussis*, and *Legionella pneumophila*, in children with MP pneumonia.

### Statistical analysis

To compare the clinical features and laboratory findings between children with positive MP specific IgM titers and those with negative MP specific IgM titers, a chi-square test or Fisher’s exact test for categorical variables or a *t*-test or Mann–Whitney U test for continuous variables were used as appropriate. Pearson’s correlation analysis was performed to identify the correlation between MP-specific IgM titers and laboratory findings with clinical features in children with positive MP-specific IgM titers. Logistic regression analysis was performed to identify factors associated with a positive MP-specific IgM titers. Adjustment was made for age, sex, macrolide resistance of MP, and duration between symptom onset and hospital visit due to MP pneumonia. All statistical analyses were performed using IBM SPSS Statistics ver. 24.0 (IBM Co., Armonk, NY, USA) software. *P*-values < 0.05 were considered statistically significant.

## Results

### Characteristics of the study population

The mean age of the participants was 6.0 ± 3.8 years (range, 0–17 yrs) and 49.0% (n = 76) of the participants were male (Table 1). At the time of admission, 76.1% (n = 118) of the children showed positive MP-specific IgM titers, whereas 23.9% (n = 37) of the children showed negative MP-specific IgM titers. Patients had a mean duration of 6.6 days (range, 0–20 days) between symptom onset and hospitalization in our hospital and the mean hospital duration was 9.5 ± 5.4 days (range, 0–31 days). A mean of 7.0 days (range, 0–23 days) of fever during the illness was observed in the study population. None of the patients died and none required admission to an intensive care unit and ventilator care in the present study. Among the 73 patients who underwent pneumobacter PCR test, 27 were identified to be coinfected with bacterial pathogens (*Hemophilus influenzae* in 11 patients; *Streptococcus pneumoniae* in 8 patients; both *H. influenzae* and *S. pneumoniae* in 8 patients).

**Table 1:** A comparison of the characteristics between children with a positive for MP-specific IgM titer at the time of admission and those with a negative MP-specific IgM titer at the time of admission due to MP pneumonia

Variables, mean ± SD or n (%)	Children positive for MP-specific IgM	Children negative for MP-specific IgM	Total	*P* value
n	118	37	155	NA
Male, n (%)	55/118 (46.6)	21/37 (56.8)	76/155 (49.0)	0.281
Age at diagnosis of MP pneumonia, years	5.8 ± 3.5	6.9 ± 4.8	6.0 ± 3.8	0.176
Duration between symptom onset and admission, days	7.4 ± 3.6	4.0 ± 3.4	6.6 ± 3.8	< 0.001
Total duration of fever during the illness, days	7.6 ± 7.1	5.0 ± 4.1	7.0 ± 6.6	0.021
Total hospitalization duration, days	9.9 ± 5.5	7.9 ± 4.6	9.5 ± 5.4	0.047
Development of any extra-pulmonary symptoms, n (%)	34/118 (28.8)	2/37 (5.4)	36/155 (23.2)	0.003
Pleural effusion, yes, n (%)	21/118 (17.8)	4/37 (10.8)	25/155 (16.1)	0.313
Hemoptysis, yes, n (%)	1/118 (0.8)	3/37 (8.1)		0.015
Oxygen need, yes, n (%)	10/118 (8.5)	0/37 (0.0)	10/155 (6.5)	0.067
Respiratory virus co-infection, yes, n (%)	53/118 (44.9)	12/37 (32.4)	65/155 (41.9)	0.179
Positive results on pneumobacter PCR, n (%)	21/61 (34.4)	6/12 (50.0)	27/73 (337.0)	0.307
Respiratory virus co-infection or positive results on pneumobacter PCR, n (%)	63/118 (53.4)	15/37 (40.5)	77/155 (49.7)	0.173
Macrolide-resistant MP	92/110 (83.6)	25/36 (69.4)	117/146 (80.1)	0.403
Response to stepwise treatment for MP pneumonia, n (%)				0.042
Good response	40/118 (33.9)	19/37 (51.4)	59/155 (38.1)	
Slow response	59/118 (50.0)	18/37 (48.6)	77/155 (49.7)	
No response	14/118 (11.9)	0/37 (0.0)	14/155 (9.0)	
Progression	5/118 (4.2)	0/37 (0.0)	5/155 (3.2)	
Trend *P*				0.007
Severity of pneumonia based on chest radiography				0.149
Mild	12/118 (10.2)	7/37 (18.9)	19/155 (12.3)	
Moderate	75/118 (63.6)	25/37 (67.6)	100/155 (64.5)	
Severe	31/118 (26.3%)	5/37 (13.5%)	36/155 (23.2)	

IgM: immunoglobulin M; MP: *Mycoplasma pneumoniae*; n: number; NA: not applicable; PCR: polymerase chain reaction; SD: standard deviation

### Comparison of clinical characteristics between children with a positive MP-specific IgM titer and those with a negative MP-specific IgM titer

The duration between symptom onset and admission (7.4 ± 3.6 days vs. 4.0 ± 3.4 days, *P* < 0.001) and total duration of symptoms during the illness due to MP pneumonia (7.6 ± 7.1 day vs. 5.0 ± 4.1 days, *P* = 0.021) was longer in children with a positive MP-specific IgM titer than those with a negative MP-specific IgM titer (Table 1). There was no significant difference in the prevalence of respiratory virus co-infection and positive results for pneumobacter PCR between positive MP-specific IgM titer and negative MP-specific IgM titer groups. In terms of responses to stepwise treatment for MP pneumonia, the proportion of slow response, no response, and progression was higher in children with positive MP-specific IgM titers than in those with negative MP-specific IgM titers. In addition, the development of extra-pulmonary symptoms of MP pneumonia was more common in children with a positive MP-specific IgM titer than in those with a negative MP-specific IgM titer (28.8% vs. 5.4%). However, there was no significant difference in the prevalence of macrolide resistance of MP between the two groups.

### Comparison of clinical features by mean duration from symptom onset to hospitalization

When the study population was divided into two groups according to the mean duration from symptom onset to admission, children hospitalized after 7 days from symptom onset showed significantly longer duration of fever during the illness and higher prevalence of development of any extra-pulmonary symptoms and macrolide resistance of MP, compared to those hospitalized within 7 days from symptom onset to admission (Table 2).

**Table 2:** Comparison of clinical features by mean duration from symptom onset to hospitalization

Variables, mean ± SD or n (%)	Within 6 days from symptom onset to hospitalization	More than 7 days from symptom onset to hospitalization	*P* value
n	83	71	NA
Male, n (%)	44/83 (53.0)	31/71 (43.7)	0.247
Age at diagnosis of MP pneumonia, years	6.1 ± 4.2	5.9 ± 3.4	0.757
Total duration of fever during the illness, days	5.2 ± 7.6	9.1 ± 4.6	< 0.001
Total hospitalization duration, days	9.0 ± 5.3	10.1 ± 5.3	0.200
Development of any extra-pulmonary symptoms, n (%)	10/83 (12.0)	26/71 (36.6)	< 0.001
Pleural effusion, yes, n (%)	9/83 (10.8)	16/71 (22.5)	0.050
Oxygen need, yes, n (%)	4/83 (4.8)	6/71 (8.5)	0.362
Respiratory virus co-infection, yes, n (%)	30/83 (36.1)	35/71 (49.3)	0.100
Positive results on pneumobacter PCR except MP, n (%)	13/32 (40.6)	14/41 (34.1)	0.569
Macrolide resistant MP, n (%)	58/80 (72.5)	59/66 (89.4)	0.011
Response to stepwise treatment for MP pneumonia, n (%)			0.657
Good response	34/83 (41.0)	24/71 (33.8)	
Poor response	49/83 (59.0)	47/71 (66.2)	
Severity of pneumonia based on chest radiography			0.089
Mild	12/83 (14.5)	6/71 (8.5)	
Moderate	57/83 (68.7)	43/71 (60.6)	
Severe	14/83 (16.9)	22/71 (31.0)	

MP: *Mycoplasma pneumoniae*; n: number; NA: not applicable; PCR: polymerase chain reaction; SD: standard deviation

### Comparison of laboratory findings between the children with a positive MP-specific IgM titer and those with a negative MP-specific IgM titer

The mean white blood cell levels (WBC, 9897 ± 4940 µ/L vs. 7130 ± 2174 µ/L, *P* < 0.001) and lactate dehydrogenase (LDH, 865.7 ± 34.6 U/L vs. 612.7 ± 21.4 U/L, *P* < 0.001) levels were significantly higher in children with a positive MP specific IgM titer than in those with a negative MP-specific IgM titer (Table 3). In addition, the mean levels of aspartate aminotransferase (AST, 47.9 ± 3.8 IU/L vs. 36.9 ± 2.5 IU/L, *P* = 0.018) and alanine aminotransferase (ALT, 38.1 ± 4.1 IU/L vs. 19.7 ± 2.3 IU/L, *P* < 0.001), which indicate liver function, were higher in children with a positive MP-specific IgM titer than in those with a negative MP-specific IgM titer.

**Table 3:** Comparison of laboratory findings between children with MP pneumonia positive for MP-specific IgM and those negative for MP-specific IgM at the time of admission due to MP pneumonia

Variables	Children positive MP-specific IgM, mean ± SD	Children negative MP-specific IgM, mean ± SD	*P* value
WBC, /µL	9897 ± 4940	7130 ± 2174	< 0.001
Neutrophil (%)	63.9 ± 14.4	60.7 ± 16.2	0.254
Lymphocyte (%)	24.8 ± 11.2	28.0 ± 14.1	0.154
Eosinophil (%)	2.1 ± 0.2	1.1 ± 0.2	0.002
Monocyte (%)	8.2 ± 0.3	9.0 ± 0.7	0.252
CRP, mg/dL	3.2 ± 0.5	3.1 ± 0.5	0.896
ESR, mm/h	37.4 ± 2.3	33.5 ± 2.4	0.237
LDH, U/L	865.7 ± 34.6	612.7 ± 21.4	< 0.001
AST, IU/L	47.9 ± 3.8	36.9 ± 2.5	0.018
ALT, IU/L	38.1 ± 4.1	19.7 ± 2.3	< 0.001
Albumin, g/dL	5.3 ± 0.1	5.2 ± 0.2	0.584
Procalcitonin, ng/mL	0.2 ± 0.0	0.4 ± 0.2	0.490

ALT: alanine aminotransferase; AST: aspartate aminotransferase; CRP: C-reactive protein; ESR: erythrocyte sedimentation rate; IgM: immunoglobulin M; LDH: lactate dehydrogenase; MP: *Mycoplasma pneumoniae*; PIBO: post-infectious bronchiolitis obliterans; SD: standard deviation; WBC: white blood cells

### Correlation between MP-specific IgM titers and clinical features and laboratory findings among children with positive MP-specific IgM titers at the time of admission in children hospitalized with MP pneumonia

**Fig. 1:**
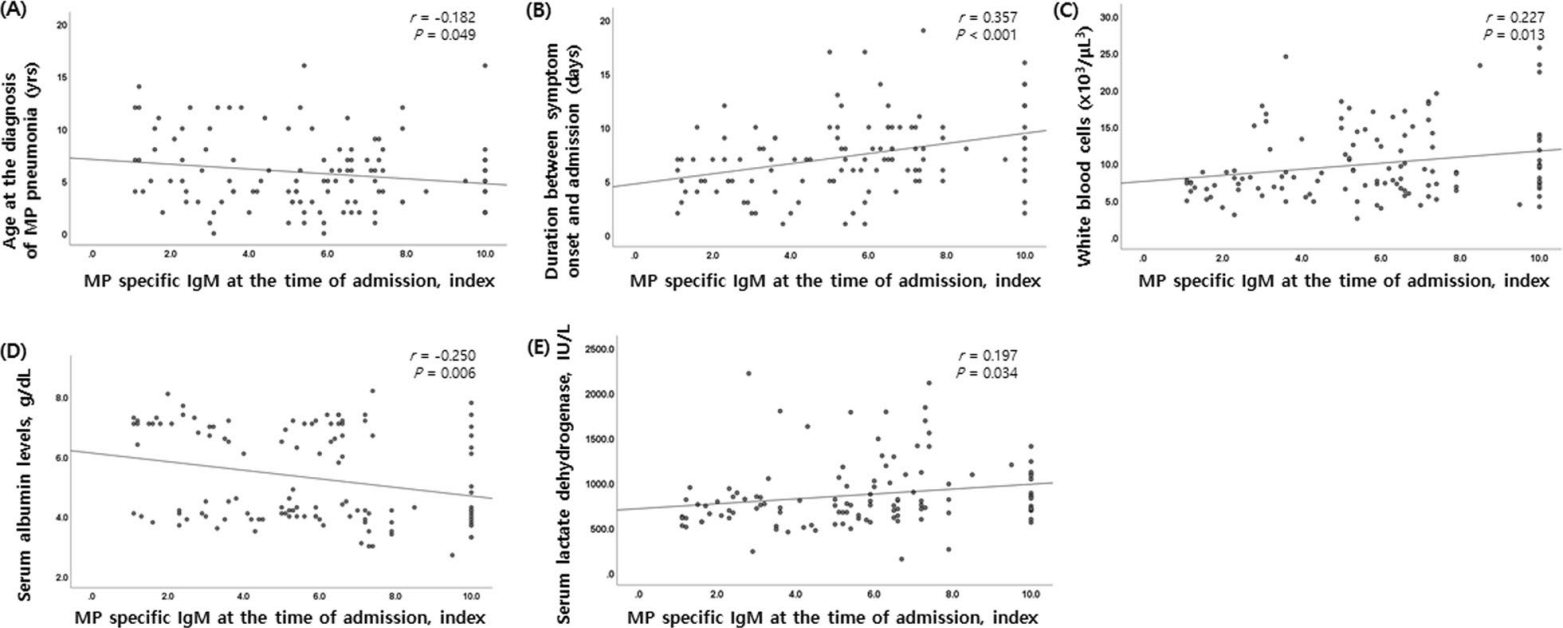
Correlation between a MP-specific IgM titer at the time of admission in children hospitalized with MP pneumonia with a positive MP-specific IgM and clinical features with laboratory findings. **A** Age at the diagnosis of MP pneumonia. **B** Duration between symptom onset and admission. **C** White blood cell counts. **D** Serum albumin levels. **E** Serum lactate dehydrogenase levels. IgM: immunoglobulin M; MP: *Mycoplasma pneumoniae*

In children with a positive MP-specific IgM titer at the time of admission due to MP pneumonia, the MP-specific IgM titer was significantly negatively correlated with age at the diagnosis of MP pneumonia (*P* = 0.049) and with serum albumin levels at the time of admission (*P* = 0.006; Fig. 1). The MP-specific IgM titer was significantly positively correlated with the duration between symptom onset and admission (*P* < 0.001), WBC count (*P* = 0.013), and serum LDH levels (*P* = 0.034). However, total duration of symptoms during the illness due to MP pneumonia did not significantly correlate with the MP-specific IgM titer titer (data not shown).

### Factors associated with a positive MP-specific IgM titer in children hospitalized due to MP pneumonia

Logistic regression analysis results indicated that a longer duration between symptom onset and admission (adjusted odds ratio [aOR] 1.47, 95% confidence interval [CI] 1.24–1.75) and longer symptom duration during the entire illness period due to MP pneumonia (aOR 1.15, 95% CI 1.02–1.30) was significantly associated with a positive MP-specific IgM in children with MP pneumonia (Table 4). Concerning laboratory findings, WBC (aOR 1.23, 95% CI 1.07–1.41) and serum LDH (aOR 1.00, 95% CI 1.00–1.01) levels were found to be significantly associated with positive MP-specific IgM titers in children with MP pneumonia. In addition, a higher extent of pneumonic infiltration on chest radiography involving > 50% of the total lung volume (aOR 4.68, 95% CI 1.12–19.55), and development of extra-pulmonary manifestations (aOR 9.16, 95% CI 1.96–42.81) were associated with positive MP-specific IgM titers in children with MP pneumonia, when those with negative MP-specific IgM titers were considered as a reference group. However, respiratory virus co-infection and positive results of pneumobacter PCR were not associated with positive MP-specific IgM titers in children with MP pneumonia, when a negative MP-specific IgM titer group was considered as a reference.

**Table 4:** Factors associated with a positive MP-specific IgM at the time of admission in children hospitalized due to MP pneumonia

Variables	OR	aOR^a^ (95% CI)	*P* value
Age, years	0.926 (0.84–1.02)	3.64 (0.38–34.54)	0.261
Duration between symptom onset and admission, days	1.46 (1.24–1.73)	1.47 (1.24–1.75)	< 0.001
Total duration of fever during illness, days	1.16 (1.03–1.30)	1.15 (1.02–1.30)	0.019
Total hospitalization duration, days	1.09 (0.99–1.19)	1.08 (0.98–1.18)	0.107
Development of any extra-pulmonary symptoms	7.08 (1.61–31.10)	9.16 (1.96–42.81)	0.005
Hemoptysis	0.10 (0.01–0.96)	0.13 (0.01–1.45)	0.097
White blood cells, /µL	1.23 (1.07–1.40)	1.23 (1.07–1.41)	0.003
Serum lactate dehydrogenase, IU/L	1.00 (1.00-1.01)	1.00 (1.00-1.01)	0.001
Serum albumin, g/dL	1.07 (0.83–1.37)	1.08 (0.84–1.39)	0.558
Respiratory virus co-infection, yes, n (%)	1.70 (0.78–3.70)	1.36 (0.59–3.14)	0.466
Positive results on pneumobacter PCR except MP, n (%)	0.56 (0.16–1.96)	0.47 (0.11–1.98)	0.301
Respiratory virus co-infection or positive results on pneumobacter PCR except MP, n (%)	1.62 (0.77–3.44)	1.40 (0.63–3.10)	0.414
Response to stepwise treatment in MP pneumonia			
Good response	Ref	Ref	
Poor response^b^	2.06 (0.97–4.35)	1.87 (0.84–4.18)	0.128
Severity of MP pneumonia based on the extent of pneumonic lesion on chest radiography at the time of admission			
Mild	Ref	Ref	
Moderate	1.75 (0.62–4.93)	2.17 (0.72–6.57)	0.171
Severe	3.62 (0.96–13.64)	4.68 (1.12–19.55)	0.034

aOR: adjusted odd ratios; IgM: immunoglobulin M; MP: *Mycoplasma pneumoniae*; OR: odds ratio; PCR: polymerase chain reaction; Ref: reference

^a^*Adjusted for age, sex, macrolide resistance of MP, and duration between symptom onset and performance date of MP-specific IgM test

^b^*Poor response includes slow response, no response and progression to stepwise treatment for MP pneumonia

## Discussion

This study identified differences in the characteristic features of children with positive MP-specific IgM titers and those with negative MP-specific IgM titers at the time of hospital admission due to MP pneumonia. Factors associated with a positive MP-specific IgM titer in children with MP pneumonia were also determined. The results of the present study would be useful to predict the clinical course and clinical outcomes in children with MP pneumonia, based on the results of a MP-specific IgM titer at the time of MP pneumonia diagnosis.

MP infection is usually confirmed using serologic tests, such as MP-specific IgM titers, and/or a PCR test. Although PCR analysis is highly sensitive and is used as a reference diagnostic method for MP detection, a PCR test cannot be always performed whenever the specimens are obtained due to high cost, time-consuming sample pretreatment, and the need for skilled technical ability [12]. In addition, a PCR assay and an MP-specific immunoglobulin G (IgG) results can lead to misdiagnosis of a current MP infection due to its long-term positivity in MP carriers [13, 14]. A false negative result in the early phase of MP infection also makes the diagnosis of MP infection challenging. Currently, a positive MP-specific IgM titer, especially a high titer, with a PCR assay combined with patient history, symptoms, physical examinations, and/or chest radiography findings is most commonly used to diagnose MP infection, although no single available test is reliable for the diagnosis of MP infection [15]. Aside from the diagnosis of MP infection, the clinical significance of a MP-specific IgM titer has not been studied. Investigation of the differences in clinical features and laboratory findings between children with positive MP-specific IgM titers and those with negative MP-specific IgM titers at the time of MP pneumonia diagnosis might provide an important significance of MP-specific IgM titer other than for the diagnosis of MP infection.

Based on the results of the present study, a longer duration of symptoms prior to visiting the hospital was associated with a positive-MP specific IgM titer, which is understandable as it takes time, usually approximately 1 week, for the seroconversion of MP-specific IgM titer after MP infection [7]. Similar patterns were observed in the total duration of fever during the illness. However, there was no significant association between total duration of hospitalization and MP-specific IgM titers, partially because the total duration of hospitalization may be determined by several factors, including subjective symptoms and objective radiologic findings with resolution of complications. The previous studies showed that MP load in MP pneumonia was associated with severe clinical course [16–18]. Based on the previous and present studies, MP load and MP-specific IgM titers were associated with clinical manifestations in MP pneumonia, which suggests the need for studies on the association between MP-specific IgM titers and MP load in MP pneumonia. Since we did not measure MP load in the present study, we could not investigate these associations. Future studies on these issues would be helpful for early diagnosis and prediction of the clinical course of MP pneumonia.

A positive MP-specific IgM is also associated with higher disease burden due to MP pneumonia, reflected in greater involvement on chest radiography findings and a longer duration of fever during the total illness with increased LDH levels. Notably, a positive MP-specific IgM has been associated with the development of extra-pulmonary manifestations during the illness and elevated serum LDH levels, which might suggest more activated immune responses in these patients [19], when compared with those with negative MP-specific IgM titers. The negative association between hemoptysis and a positive MP-specific IgM titer might be related to the early hospital visits due to hemoptysis in children with MP pneumonia. Although respiratory virus co-infection and/or bacterial co-infection can cause a severe clinical course [20, 21], coinfection with respiratory virus or bacteria in MP pneumonia was not associated with positive MP-specific IgM titer in the present study.

The influence of age on the MP-specific IgM titer in MP infection has not been fully elucidated. The previous studies have suggested that age might affect the likelihood of a positive MP specific IgM [15, 22]. In this study, there was a significantly negative correlation between age at the time of MP pneumonia diagnosis and MP-specific IgM titers among children with a positive MP-specific IgM titer, although there was no significant association between age and a positive MP-specific IgM titer at the time of diagnosis of MP pneumonia even after adjustment for the duration between symptom onset and time at MP-specific IgM. Future studies on age-related antibody response and antibody production with host protection in MP infection are needed to decrease the disease burden related to MP infection.

In terms of responses to stepwise treatment for MP pneumonia, the proportion of poor response, defined as a combination of slow response, no response, and progression, was higher with a significantly increasing trend in children with positive MP-specific IgM titers, compared with those with negative MP-specific IgM titers. There are some debates on whether macrolide resistance of MP may affect the response to the stepwise treatment for MP pneumonia [23–25] and there have been no studies on the effects of macrolide resistance of MP on MP-specific IgM titer in MP pneumonia. In the present study, there was no significant association between response to the stepwise treatment in MP pneumonia and positive MP-specific IgM titers, even when the macrolide resistance of MP was adjusted with age, sex, and duration between symptom onset and performance date of MP-specific IgM test.

The LDH level in MP pneumonia has been considered an important predictor of refractory MP pneumonia [26, 27]. When combined with serum LDH levels and the response to stepwise treatment for MP pneumonia, a positive MP-specific IgM titer at the time of diagnosis of MP pneumonia in children might suggest higher possibilities of refractory MP pneumonia [27]. Furthermore, when considering the duration between symptom onset and seroconversion of a MP-specific IgM titer, our results suggest that the early introduction of stepwise treatment for MP pneumonia might be important to improve the response to treatment for MP pneumonia.

The positive association between a higher extent of pneumonic infiltration on chest radiography findings and a positive MP-specific IgM titer might suggest a higher degree of disease extent with time in MP pneumonia, especially when combined with serum LDH levels. The positive MP-specific IgM titers at the time of diagnosis of MP pneumonia suggest an exaggerated immune response with higher disease burden due to MP pneumonia progression compared with a negative MP-specific IgM titer. Therefore, early diagnosis of MP pneumonia in children using reliable diagnostic tools enable the improvement of the prognosis in MP pneumonia in children.

This study had several limitations. The number of study participants was relatively small. The gold standard use of serologic tests for a diagnosis of MP infection is to confirm a ≥ 4 times increase in the MP-specific IgG titer 2–3 weeks after the first measurement; however, we did not measure the MP-specific IgG titer to diagnose MP infection in this study due to its low clinical usefulness for early diagnosis of MP infection [28]. Nevertheless, the results of the present study provide a novel insight into the clinical usefulness of MP-specific IgM titers in children with MP pneumonia through a comparison of the clinical and laboratory findings between children with positive MP-specific IgM titers and those with negative MP-specific IgM titers at the time of admission due to MP pneumonia. Respiratory virus coinfection and pneumobacter PCR was performed in a part of the study population. Further large-scale studies are needed to identify the association between respiratory co-infection and positive MP-specific IgM titers in MP pneumonia.

In conclusion, a positive MP-specific IgM titer at the time of diagnosis of MP pneumonia may partially suggest an exaggerated immune response with higher disease burden due to progression of MP pneumonia, compared to children with a negative MP-specific IgM titer.

## Data Availability

The datasets generated and/or analysed during the current study are not publicly available due to personal identifiable information, but are available from the corresponding author on reasonable request.

## Abbreviations

aOR: Adjusted odds ratio
AST: Aspartate aminotransferase
ALT: Alanine aminotransferase
CRP: C-reactive protein
IgM: Immunoglobulin M
LDH: Lactate dehydrogenase
MP: *Mycoplasma pneumoniae*
NA: Not applicable
PCR: Polymerase chain reaction
Ref.: Reference
